# Evidence for an Intrathecal Immunoglobulin Synthesis by Kappa Free Light Chains in Neurological Patients with an Isolated Band in Isoelectric Focusing

**DOI:** 10.3390/biomedicines10092202

**Published:** 2022-09-06

**Authors:** Bastian Weiss, Alexander Pichler, Anna Damulina, Arabella Buchmann, Sonja Hochmeister, Thomas Seifert-Held, Christian Enzinger, Juan-Jose Archelos, Michael Khalil

**Affiliations:** 1 Department of Neurology, Medical University of Graz, AT-8036 Graz, Austria; 2 Neurology Biomarker Research Unit, Department of Neurology, Medical University of Graz, AT-8036 Graz, Austria

**Keywords:** immunoglobulin kappa-chains, isoelectric focusing, neuroinflammatory diseases, multiple sclerosis, human, biomarker, cerebrospinal fluid, central nervous system

## Abstract

The gold standard for detecting intrathecal immunoglobulin synthesis is the determination of the oligoclonal band (OCB) in the cerebrospinal fluid (CSF) using isoelectric focusing (IEF). Controversy still exists regarding the significance of an isolated band in the CSF. A highly promising alternative method for the assessment of intrathecal inflammation is the quantification of kappa free light chains (k-FLC). Our aim was to evaluate the clinical significance of quantitative k-FLC in patients with an isolated band in the CSF. Using the Human Kappa Freelite Mx Kit on a turbidimetric Optilite^®^, we quantified the k-FLCs in paired CSF and serum samples in 47 patients with a single band in IEF. We classified patients into 27× inflammatory neurological disorders (IND), 2× peripheral inflammatory neurological disorders (PIND), 9× non-inflammatory neurological disorders (NIND) and 9× symptomatic controls (SC) based on their medical diagnosis. k-FLC were below the lower measurement limit of the analyser (LML) in all SC and PIND, as well as in 8 out of 9 NIND and 11 IND. Only 1 NIND and 16 IND were above the LML, and of these, only 14 IND were above the upper discrimination limit (Qlim). A neuroinflammatory nature of the diseases can be indicated in many cases by positive k-FLC in patients with an isolated band in IEF. The measurement of k-FLC can support the diagnosis of neurological diseases if they are included in the routine work-up.

## 1. Introduction

The detection of intrathecal immunoglobulin synthesis in neuroimmunological diseases can be performed using various laboratory analytical methods. Nevertheless, qualitative interpretation of isoelectric focusing (IEF) is still considered as the gold standard [1,2]. IEF is followed by silver staining or immunoblotting, whereby the presence of cerebrospinal fluid (CSF) restricted oligoclonal bands (OCB) indicates a central nervous system-specific B cell activation, which is a hallmark of various neuroimmunological diseases, including multiple sclerosis (MS) [1,2,3]. A finding with two (additional) bands or more in CSF is considered positive according to consensus definition [1,2,4]. Borderline findings in laboratory analyses, namely an isolated CSF-restricted band, often pose great challenges to clinicians due to the clinical significance of one isolated band still being a matter of debates [5,6,7].

As a relatively labour-intensive and costly procedure, IEF has a long turnaround time of 4–5 h. Furthermore, due to visual interpretation of the results, rater-dependent bias can occur, which has served as a motivational factor to search for easier to standardise and faster laboratory options [8]. An alternative to IEF is the quantification of kappa free light chains (k-FLC) in the CSF [9,10,11,12]. k-FLC are produced by B/plasma cells and are present in a free form in serum and CSF, resulting from excess production compared with the heavy chains. Hence, these k-FLC are measurable similar to intact immunoglobulins despite their shorter half-life [13]. Measured turbido- or nephelometrically with low laboratory effort, k-FLC have shown comparable sensitivity and specificity in the detection of neuroimmunological diseases such as MS in recent studies [9,10,11,12].

However, up to now, the performance of k-FLC in borderline findings in the IEF, in the case of one isolated band, has not sufficiently been investigated. There is only one recent study showing that the measurement of k-FLC can confirm intrathecal inflammation, and a lack of k-FLC almost always indicates a non-inflammatory aetiology [14]. The aim of this work is to determine the clinical significance of k-FLC in the presence of an isolated band in IEF and therefore to support clinicians’ decision making in cases of suspected intrathecal immunoglobulin synthesis in neuroimmunological diseases and borderline findings in IEF.

## 2. Materials and Methods

### 2.1. Laboratory Assay

Paired serum and CSF samples, collected from patients at the Department of Neurology of the Medical University of Graz between 2006 and 2019, were stored at −80° Celsius in the CSF laboratory of the Department or in the Biobank of the Medical University of Graz. Pre-freezing routine diagnostics were performed using a Beckman Coulter Image 800 analyser (Beckman Coulter Inc., Brea, CA, USA) (for albumin, IgG, IgA and IgM) and Fuchs Rosenthal Counting Chambers (for white blood cells). In addition, OCBs were qualitatively determined using IEF followed by silver staining or immunoblotting and assessed, as well as descriptively reported by experienced raters (SH, JJA, TSH and MK). k-FLC were quantified from the paired serum and CSF samples using the Human Kappa Freelite Mx Kit (The Binding Site Group Ltd., Birmingham, UK) on the Optilite^®^ turbidimeter. The lower measuring limit of the analyser for k-FLC was 0.28 mg/L.

All laboratory analyses were carried out at the CSF laboratory at the Department of Neurology of the Medical University of Graz and working methods were evaluated by internal and external quality management. Control measurements were carried out on the analysers according to the manufacturer’s guidelines (e.g., once per day on the Optilite^®^, with two different concentrations). We followed international consensus statements on the processing and analysis of CSF [15].

### 2.2. Patients

Exclusion criteria for the study were IEF findings that were described as clearly positive, i.e., had more than one band in the CSF. Additionally, IEFs which were described as clearly negative, i.e., polyclonal in the CSF or had the same number of bands in the serum as in the CSF, were also excluded. Only the IEFs that contained signal words in the descriptive reports (e.g., “a thin/fine band”, “an additional band” or “an isolated band”) were considered for the study. Furthermore, the descriptive reports that indicated a single band in the CSF were also evaluated in a re-examination of the IEFs. This re-examination was performed by experienced raters (SH and MK). Only the IEFs that were also rated with a single band in the re-examination were included for the calculations, on condition that the patients were at least 18 years old and the clinical documentation was complete.

Patients’ clinical diagnoses were assessed using the clinic’s electronic work-up and documentation programme and categorised into inflammatory neurological disease (IND, with a cut off value of white blood cells in the CSF for a pleocytosis of more than 4 cells), peripheral inflammatory neurological disease (PIND), non-inflammatory neurological disease (NIND) and symptomatic controls (SC) based on Teunissen et al. (2013) [16]. The diagnosis of MS was made using the recent version of the McDonald criteria [1].

### 2.3. Statistical Analysis

SPSS 27.0 (IBM Co., Armonk, NY, USA) and RStudio (R version 3.5.1 2021-09-20) were used for statistical analyses and graphical visualisations. To test for a normal distribution of the data, Kolmogorov–Smirnov analysis was performed. Furthermore, the chi-square test was executed for group differences in qualitative outcomes while the Mann–Whitney-U test for two samples, as well as Kruskal–Wallis test for more than two samples for quantitative, non-parametric outcomes. Statistical significance was assumed from a *p*-value of < 0.05. For multiple testing, the significance level was adapted by the Bonferroni correction.

Sensitivity and specificity, as well as positive predictive value (PPV) and negative predictive value (NPV), were calculated by forming two groups (IND and PIND versus NIND and SC), meaning all the values for the detection of the IND/PIND group. Previously, it was checked whether there were statistically significant differences between NIND and SC regarding routine laboratory parameters. Sensitivity was computed as (true-positive/(true-positive + false-negative)), specificity as (true-negative/(true-negative + false-positive)). The PPV was computed as (true-positive/(true-positive + false-positive)) and the NPV as (true-negative/(true-negative + false-negative)).

Threshold values of k-FLC for the presence of intrathecal immunoglobulin synthesis were calculated using the hyperbolic formula according to Reiber et al. (2019) [17] to allow comparability with the previous study [14]. Furthermore, CSF/serum quotients of the routine parameters and k-FLC, as well as the k-FLC index by correction for the albumin quotient (k-FLC index = k-FLC quotient/albumin quotient), were calculated.

### 2.4. Ethical Standards

The study was conducted according to the guidelines of the Declaration of Helsinki. The Ethics Committee of the Medical University of Graz approved the study protocol and design (32-029 ex 19/20).

## 3. Results

From 2006 to 2019, a total of 4082 analyses from paired serum and CSF samples were performed. In the course of these analyses, 1798 IEFs were made, whereby 88 potential IEFs with a single band (4.89% of all IEFs made) were identified by screening the descriptive findings. After re-examination of these IEFs, 47 pairs with one isolated band (2.61% of all IEFs made), for which samples and complete clinical data were also available and the patients were over 18 years of age, were included in the study for measurement of k-FLC.

**Figure 1:**
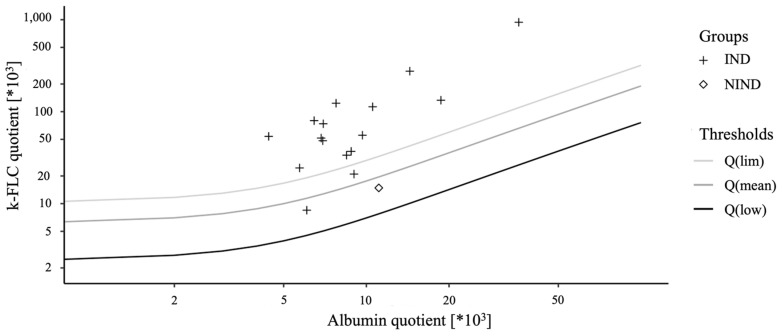
Data of the cohort in a double logarithmic kappa free light chains (k-FLC) Reibergram. Data points of the k-FLC measurement results plotted on a double logarithmic scale, corresponding to the Reibergram. Q(lim) represents the upper discrimination line, Q(mean) the middle and Q(low) the lower. IND: inflammatory neurological disease; NIND: non-inflammatory neurological disease.

Based on the patients’ diagnoses and further laboratory analyses, these samples could be classified into 27× IND, 2× PIND, 9× NIND and 9× SC. The measurement of the k-FLC showed that all SC and PIND were below the lower measurement limit of the analyser (LML), as were 8 out of 9 NIND and 11 IND. Only 14 out of 16 IND above the LML were also above the upper discrimination limit (Qlim), which would indicate intrathecal immunoglobulin synthesis. One NIND was above the LML, but below Qlim (Table 1 and Figure 1). This resulted in a sensitivity for the IND/PIND group of 48.3% and a specificity of 100% for the detection of intrathecal immunoglobulin synthesis in this study population. Consequently, the PPV and NPV for this cohort calculated are 100% and 54.6%, respectively. Considering the sensitivity and NPV of the IND group without PINDs, the values would be minimally better (sensitivity = 51.9%, NPV = 60.6%).

The diagnoses of the IND above Qlim included 1× MS, 1× clinically isolated syndrome, 1× neuromyelitis optic spectrum disorder, 1× transverse myelitis, 3× retrobulbar neuritis, 5× meningitis/encephalitis, as well as 1× herpes zoster ophthalmicus and 1× cerebral neoplasia with signs of inflammation in the laboratory. The IND group diagnoses below Qlim included 1× MS, 1× transverse myelitis, 2× retrobulbar neuritis, 3× meningitis/encephalitis, 1× neuroborreliosis, 1× herpes zoster ophtalmicus, 1× disc herniation with myelopathy, 1× cerebral neoplasia, 1× basilar migraine, as well as 1× spinal canal stenosis, each of these with signs of inflammation in the laboratory. In one patient each, classified as PIND according to the definition, peripheral facial nerve palsy and polyradiculoneuritis were diagnosed, but both were below the LML. There were no statistically significant differences in routine laboratory parameters between INDs above Qlim versus INDs below Qlim.

As shown in Table 1, there were no significant differences in age and sex distribution among the groups (*p*-values: 0.776 and 0.627). However, there were significant variations in routine parameters, namely in the number of white blood cells (*p* = 0.001) and the CSF/serum quotients of albumin (*p* = 0.001), IgG (*p* = 0.002), IgM (*p* = 0.013) and IgA quotient (*p* = 0.004). Subgroup analyses of these laboratory parameters between IND, NIND and SC reveal statistically significant differences in white blood cells between IND and SC (*p* = 0.031) and between IND and NIND (*p* = 0.043). In addition, there were statistically significant differences in the albumin quotient between IND and SC (*p* = 0.008) and between IND and NIND (*p* = 0.034), as well as in the IgG quotient between IND and SC (*p* = 0.026) and in the IgA quotient between the same groups with a *p*-value of 0.025. Due to the small sample size of the PIND group, it was not included in the subgroup analysis.

## 4. Discussion

The evaluation of k-FLC in laboratory work-up has proven to be a useful additional parameter within the diagnosis of intrathecal immunoglobulin synthesis [8,9,10,11,12], which has also been determined to be robust against external influences such as storage, plasma exchange and common therapy in acute exacerbation in MS (depending on drug concentration) [9]. Furthermore, in the case of artificial blood contamination of the CSF, k-FLC does not increase artificially compared with the Ig indices [18].

By measuring the k-FLC, patients with an IND and an isolated band in the IEF in CSF could be detected with a sensitivity of 51.9%. Specificity was 100% because only patients with an IND were above the threshold for positive k-FLC. Similar sensitivity and specificity have been reported in past studies of patients with more than one band in IEF, where k-FLC were measured quantitatively [9,19]. In this regard, our results are also mainly consistent with the ones of a previous study on only one isolated band [14].

Although the frequency of unclear findings with an isolated band in the IEF is also relatively low in the total number of analyses performed (47 of 1798 IEF findings = 2.61%), it nevertheless appears to be important to determine whether the findings are based on intrathecal immunoglobulin synthesis or not. As is well-known, an isolated band can occur accidentally [5], or even if the CSF analysis was performed in patients in an early phase of intrathecal IgG production [7]. Even though many diseases of the IND group can also be categorised as infectious, it has already been established that k-FLC also has a high sensitivity and specificity in neuroborreliosis [20,21]. Nevertheless, further studies are needed to find out to what extent this can also be applied to other bacterial and viral infections, as there is only one study so far that has investigated k-FLC in Tick-Borne Encephalitis [22].

In regard to MS and clinically isolated syndrome, it has been shown that the measurement of k-FLC can make a valuable contribution to diagnosis, with its high sensitivity and specificity, similar to that of IEF [9,10,11,12]. Moreover, in early forms of this disease (e.g., with a single clinical attack so far), k-FLC still has respectable diagnostic value [11] and can be considered as a predictive factor for disease activity [11,23,24]. The measurement of k-FLC, used as a screening and diagnostic algorithm, can in this way not only relieve IEF-inexperienced laboratories and standardised laboratories but also reduce workload and costs [8,25].

It was also shown that 96% of patients with MS-related myelitis and 55.6% of patients with NMOSD myelitis have intrathecal inflammation, and thus k-FLC synthesis [26], which is also in line with our results. Therefore, our study clearly shows an added value of k-FLC in delineating borderline IEF findings, particularly the fact that only IND patients were above the threshold. To further validate our findings, multicentre studies investigating larger cohorts should be performed soon.

Furthermore, it is yet not completely clarified which cut-off values of k-FLC should be applied, as studies have been performed with different approaches (e.g., with a quotient, index or hyperbolic calculation) [9]. In any case, with the introduction of the Reibergram for k-FLC [17], a sound tool is available that also allows dichotomous classification (positive and negative) and takes a possible blood–brain barrier dysfunction into account [9,27]. In consideration of the aspects described above, our study emphasises the clinical significance of k-FLC quantification even in borderline findings in IEF.

## Figures and Tables

**Table 1:** Baseline characteristics and measurement results of the k-FLC according to the medical diagnosis of the patients with one isolated band in IEF.

	IND/PIND (*n* = 29)	NIND/SC (*n* = 18)	*p*-Value
age	34.0 (26.5–50.5)	32.0 (24.8–56.0)	0.776
female	14 (48.3%)	10 (55.6%)	0.627
WBC/µL	12.0 (3.5–54.5)	2.0 (1.0–4.0)	0.001 *
Qalb × 10^−3^	7.8 (5.2–11.0)	4.4 (3.6–6.6)	0.001 *
QIgG × 10^−3^	4.4 (3.2–6.2)	2.4 (2.1–4.1)	0.002 *
QIgM × 10^−3^	1.0 (0.7–3.0)	0.6 (0.3–1.3)	0.013 *
QIgA × 10^−3^	0.3 (0.3–0.4)	0.3 (0.3–0.4)	0.004 *
k-FLC serum (mg/L)	14.6 (10.9–20.6)	-	
k-FLC CSF (mg/L)	0.8 (0.6–2.2)	-	
k-FLC index	7.3 (4.2–12.3)	-	
>Q(lim)	14	0	
<Q(lim)	2	1	
<LML	13	17	

IND: inflammatory neurological diseases; PIND: peripheral inflammatory neurological diseases; NIND: non-inflammatory neurological diseases; SC: symptomatic controls; WBC: white blood cells; Qalb: albumin quotient; QIgG: immunoglobulin G quotient; QIgM: immunoglobulin M quotient; QIgA: immunoglobulin A quotient; k-FLC: kappa free light chains; CSF: cerebrospinal fluid; Q(lim): upper discrimination line; LML: lower measuring limit of the analyser. Continuous variables are given as median (interquartile range), nominal variables additionally as percentages in brackets. * *p*-Values < 0.05 indicate statistical significance.

## Data Availability

The data that support the findings of this study are available from the corresponding author upon reasonable request.
